# Effect of Telehealth Extended Care for Maintenance of Weight Loss in Rural US Communities: A Randomized Clinical Trial

**DOI:** 10.1001/jamanetworkopen.2020.6764

**Published:** 2020-06-15

**Authors:** Michael G. Perri, Meena N. Shankar, Michael J. Daniels, Patricia E. Durning, Kathryn M. Ross, Marian C. Limacher, David M. Janicke, A. Daniel Martin, Kumaresh Dhara, Linda B. Bobroff, Tiffany A. Radcliff, Christie A. Befort

**Affiliations:** 1 Department of Clinical and Health Psychology, University of Florida, Gainesville; 2 Department of Statistics, University of Florida, Gainesville; 3 Department of Medicine, University of Florida, Gainesville; 4 Department of Physical Therapy, University of Florida, Gainesville; 5 Department of Family, Youth, and Community Sciences, University of Florida, Gainesville; 6 Department of Health Policy and Management, Texas A&M University, College Station; 7 Department of Population Health, University of Kansas Medical Center, Kansas City

## Abstract

**Importance:**

Lifestyle interventions for obesity produce reductions in body weight that can decrease risk for diabetes and cardiovascular disease but are limited by suboptimal maintenance of lost weight and inadequate dissemination in low-resource communities.

**Objective:**

To evaluate the effectiveness of extended care programs for obesity management delivered remotely in rural communities through the US Cooperative Extension System.

**Design, Setting, and Participants:**

This randomized clinical trial was conducted from October 21, 2013, to December 21, 2018, in Cooperative Extension Service offices of 14 counties in Florida. A total of 851 individuals were screened for participation; 220 individuals did not meet eligibility criteria, and 103 individuals declined to participate. Of 528 individuals who initiated a 4-month lifestyle intervention, 445 qualified for randomization. Data were analyzed from August 22 to October 21, 2019.

**Interventions:**

Participants were randomly assigned to extended care delivered via individual or group telephone counseling or an education control program delivered via email. All participants received 18 modules with posttreatment recommendations for maintaining lost weight. In the telephone-based interventions, health coaches provided participants with 18 individual or group sessions focused on problem solving for obstacles to the maintenance of weight loss.

**Main Outcomes and Measures:**

The primary outcome was change in body weight from the conclusion of initial intervention (month 4) to final follow-up (month 22). An additional outcome was the proportion of participants achieving at least 10% body weight reduction at follow-up.

**Results:**

Among 445 participants (mean [SD] age, 55.4 [10.2] years; 368 [82.7%] women; 329 [73.9%] white), 149 participants (33.5%) were randomized to individual telephone counseling, 143 participants (32.1%) were randomized to group telephone counseling, and 153 participants (34.4%) were randomized to the email education control. Mean (SD) baseline weight was 99.9 (14.6) kg, and mean (SD) weight loss after the initial intervention was 8.3 (4.9) kg. Mean weight regains at follow-up were 2.3 (95% credible interval [CrI], 1.2-3.4) kg in the individual telephone counseling group, 2.8 (95% CrI, 1.4-4.2) kg for the group telephone counseling group, and 4.1 (95% CrI, 3.1-5.0) kg for the education control group, with a significantly smaller weight regain observed in the individual telephone counseling group vs control group (posterior probability >.99). A larger proportion of participants in the individual telephone counseling group achieved at least 10% weight reductions (31.5% [95% CrI, 24.1%-40.0%]) than in the control group (19.1% [95% CrI, 14.1%-24.9%]) (posterior probability >.99).

**Conclusions and Relevance:**

This randomized clinical trial found that providing extended care for obesity management in rural communities via individual telephone counseling decreased weight regain and increased the proportion of participants who sustained clinically meaningful weight losses.

**Trial Registration:**

ClinicalTrials.gov Identifier: NCT02054624

## Key Points

**Question:**

Does extended care for obesity management delivered remotely via individual or group telephone counseling in rural communities improve the maintenance of weight loss compared with an education program about weight gain prevention?

**Findings:**

In this randomized clinical trial of 445 participants who completed a lifestyle weight-loss intervention, extended care delivered remotely via individual telephone counseling decreased weight regain and increased the proportion of participants who achieved clinically meaningful weight losses compared with education alone.

**Meaning:**

These findings suggest that extended care delivered remotely via individual telephone counseling may reduce weight regain and increase the proportion of participants who achieve long-term weight reductions of at least 10%.

## Teaser

This randomized clinical trial examines the efficacy of individual or group telephone counseling vs an educational control for weight loss maintenance among individuals in rural communities in the US.

## Introduction

The burden of obesity in the US disproportionately affects rural communities. Compared with urban and suburban locales, rural areas have a greater prevalence of obesity^1,2^ and higher rates of obesity-related morbidity and mortality.^3,4^ Rural communities have less access to preventive health care services,^4,5,6^ including comprehensive lifestyle treatment for obesity—the criterion standard recommended by the US Preventive Services Task Force^7^ and multiple scientific societies.^8^ Documentation of the effectiveness of lifestyle treatment has come from efficacy trials, commonly conducted with middle-class participants from urban or suburban locales and delivered by experts from academic medical centers.^9,10,11^ Few studies have been implemented in medically underserved rural settings with local staff delivering treatment to economically disadvantaged participants, and current dissemination efforts have failed to narrow the rural-urban gap in access to weight-management services.^12^

Comprehensive lifestyle interventions can produce initial body weight reductions of 5% to 10%,^7,8,9,10,11^ a magnitude of weight loss that can yield clinically significant reductions in hypertension and hyperlipidemia and prevent the onset of type 2 diabetes.^13^ However, after treatment ends, participants typically begin to regain weight—often regaining one-third to one-half of initial weight losses within 1 year.^8,11,14^ Supplementing initial treatment with extended care programs delivered via face-to-face sessions can improve the maintenance of weight loss.^15,16,17,18^ However, in rural communities, the long distances that participants must travel to attend face-to-face sessions constitutes a major barrier to implementation.^5^ The Treatment of Obesity in Underserved Rural Settings trial^19,20^ demonstrated that providing extended care via individual telephone counseling improved the maintenance of weight loss, comparable with face-to-face sessions (and at a lower cost), compared with an education control. However, individual telephone counseling is resource intensive with respect to interventionist time. Using a conference-call format to deliver telephone counseling simultaneously to multiple participants may provide an innovative, cost-effective alternative to individual telephone counseling.^21^ In a randomized clinical trial conducted in a rural setting in 2016,^22^ lifestyle treatment delivered entirely via group telephone counseling improved long-term weight losses compared with a newsletter control group. However, that study provided participants with 2 free meal-replacements per day during initial treatment. While such an approach may increase initial weight loss, it complicates the interpretation of the effects of extended care interventions, which began at the same time that meal replacements ended. Moreover, the provision of no-cost meal replacements has limited feasibility for implementation in low-resource rural communities. To our knowledge, no trial has examined the effectiveness of group-based telephone counseling as an extended-care intervention in rural areas absent the use of study-provided meal replacements.

This study was a randomized clinical trial designed to evaluate the effectiveness of individual and group telephone extended care programs for weight-loss maintenance in rural communities compared with an education control. Treatment was delivered through the US Cooperative Extension System (CES),^23,24^ a federal-state-county partnership with offices in approximately 2900 of 3007 counties in the US. We hypothesized that extended care delivered via either individual or group telephone counseling would produce greater long-term maintenance of weight loss than an education control program.

## Methods

This study was approved by the University of Florida institutional review board, and all participants provided written informed consent. This study followed the Consolidated Standards of Reporting Trials (CONSORT) reporting guideline. The trial was conducted from October 21, 2013, to December 21, 2018, in 3 phases (trial protocol in Supplement 1). Phase 1 entailed an initial weight-loss period during which all participants received the same face-to-face, comprehensive group lifestyle intervention. A description of the study design, including phase 1 enrollment and initial weight changes, has been published elsewhere.^25^ Phase 2 involved a 12-month period during which participants received extended care according to randomized assignment. Phase 3 was a 6-month period with no participant-interventionist contact. Based on an expected Phase 1 enrollment of 540 participants, the study was powered at 0.80 (2-sided tests with Bonferroni corrections) to detect a 2.5-kg difference in weight regain between the individual telephone counseling group vs the control group and the group telephone counseling group vs the control group.

### Participants

**Figure 1.**
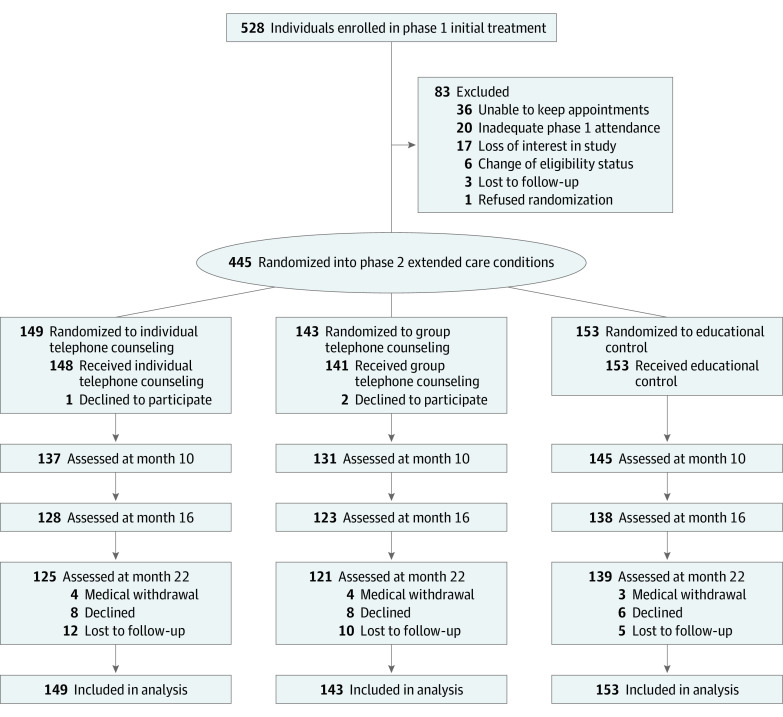
Diagram of Participant Flow

As previously reported,^25^ 851 adults from rural communities underwent screening at CES offices; 220 individuals did not meet eligibility criteria, and 103 individuals declined participation. Phase 1 participants included 528 adults^26,27^ aged 21 to 75 years with body mass index (BMI; calculated as weight in kilograms divided by height in meters squared) ranging from 30 to 45, and no medical contraindications for weight loss. Of those who initiated the intervention, 445 individuals (84%) attended at least 50% of planned sessions and thereby qualified for randomization.^25^ Figure 1 presents the diagram of participant flow.

### Health Coaches

The intervention was delivered by CES family and consumer sciences extension agents or individuals with a bachelor’s or master’s degree in nutrition, exercise science, or psychology. All interventionists were provided with training in comprehensive lifestyle treatment^11^ and the use of problem-solving counseling.^16,28^

### Phase 1

All participants completed a 16-week intervention^29,30^ delivered face-to-face in groups of 4 to 16 participants at CES offices in 14 counties of northern Florida. Intervention content addressed challenges commonly experienced by individuals from rural areas (eg, tradition of high-calorie “country” cooking and a lack of community exercise facilities).^19^ Caloric intake goals were based initially on baseline weight (ie, 1200 kcal per day for individuals weighing ≤113.6 kg; 1500 kcal per day for individuals weighing >113.6 kg) and subsequently were modified based on weight-loss progress. Participants were instructed to keep daily logs of the types and amounts of foods consumed along with corresponding caloric values and to increase planned daily walking by 3000 steps. Participants received training in the completion of food logs and were provided with a reference book listing caloric values of common foods and drinks.^31^ They were given the option of using smartphone applications or paper-and-pencil forms and were instructed to specify their caloric intake goals on their self-monitoring logs and to indicate whether these goals were achieved. During phase 1, health coaches collected self-monitoring records at each session. During phase 2, participants were provided envelopes with prepaid postage to return their self-monitoring records. Health coaches provided feedback regarding potential changes to improve adherence to caloric intake goals.

### Phase 2

The study statistician (M.J.D.), using a random number generator, assigned eligible individuals to 1 of 3 phase 2 groups. County size, county, phase 1 group, and session time were balanced during randomization. All participants received written guidelines describing how to use problem-solving strategies to deal with obstacles to the maintenance of lost weight. All participants were instructed to continue to complete self-monitoring records and to sustain their efforts to achieve caloric intake and physical activity goals. The method of interventionist contact during phase 2 differed by group, but the schedule of contacts was consistent across groups (biweekly during months 5-10 and monthly during months 11-16). At each contact point, all participants received a weight-management module delivered via email (and US mail if requested) with information and recommended behavioral activities targeting the maintenance of lost weight.^25^

#### Individual Telephone Counseling

Participants in the individual telephone counseling group called into a teleconference line for a scheduled, one-on-one, 10- to 20-minute session with their health coach. Each session included a review of progress toward goals paired with structured problem solving to address any reported challenges,^16,28^ a discussion of the weight-management module, and goal setting and the development of an action plan specifying diet and/or physical activity changes to be accomplished prior to the next session.

#### Group Telephone Counseling

Participants in the group telephone counseling group called into a teleconference line for a 60-minute group session with the health coach and other participants. These sessions entailed the same structure, components, and problem-solving focus as the individual calls.

#### Education Control Program

Participants in the education control group received 18 weight-management modules with the identical content used in the telephone groups. Education modules were delivered on the same schedule as the telephone contacts.

All individual and group sessions were audio recorded, and a sample of recordings from 10% of participants in the individual and group counseling groups were coded for treatment fidelity using a 15-point checklist. Treatment fidelity was high in the individual and group telephone counseling groups with a mean (SD) of 12.83 (1.72) in the individual telephone counseling group and 13.78 (1.96) in the group telephone counseling group.

### Phase 3

For study participants, phase 3 of the trial represented a transition to self-reliance. During months 17 through 22, there was no contact between participants and health coaches in any of the groups.

### Measures

Weight was measured with a digital scale by study staff, masked to randomization, at baseline and months 4, 10, 16, and 22. Height was measured with a stadiometer at baseline. Achievement of caloric intake goals was based on participants’ self-reported daily assessment (yes or no) recorded on the self-monitoring record. For days without a record or an incomplete record (ie, missing type and quantity of food for ≥1 meal), it was assumed that the intake goal was not met.

### Statistical Analysis

Preliminary analyses were conducted to evaluate baseline differences between groups and between-group differences in attendance and adherence during phase 2. We used a Bayesian approach and an intent-to-treat estimate for the analyses of weight and the proportion of participants achieving 5% and 10% weight losses.^32^ For the primary outcome (ie, weight change from months 4 to 22), we used the NiNBayes R package (R Project for Statistical Computing), which modeled the weights longitudinally using a Dirichlet process mixture of models.^33^ Two different assumptions regarding missing data were examined: missing at random and missing not at random. In the missing-not-at-random analysis, individuals who discontinued participation prior to month 22 were assumed to have regained weight at an mean rate of 0.3 kg per month after dropping out.^11,19^ Intermittent missingness was assumed to be partially ignorable.^34^ Inferences were based on examination of whether the 95% credible intervals (CrIs) contained 0 and were quantified by posterior probabilities (PPs). The results were highly similar under missing-at-random and missing-not-at-random assumptions; hence, only the missing-at-random results are reported. We also analyzed percentage change in body weight during months 4 to 22 by computing percentage weight changes from the models used for the primary outcome.

A causal mediational analysis^35^ was conducted to examine whether adherence to caloric intake goals mediated the association between extended care group and weight change from months 4 to 22. An assumption of sequential ignorability was made,^35^ and sex and self-reported race/ethnicity were included as possible confounders.

The analyses of weight, weight changes, and causal mediation were conducted using R statistical software. Analyses of participant characteristics, attendance, and adherence were completed using SPSS Statistics for Windows version 21.0 (IBM). *P* values were 2-sided, and statistical significance was set at α = .05. Data were analyzed from August 22 to October 1, 2019.

## Results

### Participant Characteristics

A total of 445 participants were included, the mean (SD) age of randomized participants at baseline was 55.4 (10.2) years, and 368 participants (82.7%) were women. Mean (SD) weight at baseline was 99.9 (14.6) kg, and their mean (SD) BMI was 36.(3.7). Among these, 149 participants (33.5%) were randomized to individual telephone counseling, 143 participants (32.1%) were randomized to group telephone counseling, and 153 participants (34.4%) were randomized to the email education control. There were no significant between-group differences in baseline weight or phase 1 percentage weight change or weight loss (mean [SD], 8.3 [4.9] kg). The sample included 329 participants (73.9%) who self-identified as non-Hispanic white, and 242 participants (54.4%) had at least 12 years of education. Among 417 participants who reported annual household incomes, 210 participants (50.4%) indicated income less than $50 000. No significant between-group differences in demographic characteristics were observed at baseline (Table 1).

**Table 1.** Participant Baseline Characteristics by Group

Characteristic	No. (%)		
	Individual telephone counseling (n = 149)	Group telephone counseling (n = 143)	Education control (n = 153)
Age, mean (SD), y	55.9 (10.2)	55.4 (9.8)	54.8 (10.7)
BMI, mean (SD)	36.3 (3.9)	37.0 (3.6)	36.1 (3.6)
Sex			
Women	126 (84.6)	118 (82.5)	124 (81.0)
Men	23 (15.4)	25 (17.5)	29 (19.0)
Race/ethnicity			
Non-Hispanic white	112 (75.2)	102 (71.3)	114 (74.5)
Non-Hispanic black	28 (18.8)	25 (17.5)	29 (19.0)
Hispanic	6 (4.0)	7 (4.9)	6 (3.9)
Other	3 (2.0)	9 (6.3)	4 (2.6)
Education level			
≤High school	85 (57.0)	74 (51.7)	79 (51.6)
Associate’s degree	16 (10.7)	21 (14.7)	17 (11.1)
≥Bachelor’s degree	48 (32.2)	48 (33.6)	57 (37.3)
Household income level			
<$50 000	70 (47.0)	65 (35.5)	75 (47.0)
≥$50 000	68 (45.6)	71 (49.7)	68 (44.4)
Unknown or refused	11 (7.4)	7 (4.9)	10 (6.5)

Abbreviation: BMI, body mass index (calculated as weight in kilograms divided by height in meters squared).

### Retention, Attendance, and Adherence

Among 445 participants, 385 participants (86.5%) attended the 22-month assessment, and there were no significant between-group differences in retention. During phase 1, participants had a mean (SD) attendance rate of 80.1% (22.6%). During phase 2, a higher attendance rate was observed among individual vs group telephone counseling participants (mean [SD], 66.9% [27.5%] vs 53.9% [27.6%]; *P* < .001). Participants in the individual telephone counseling group had more days with completed self-monitoring records than participants in the control group (mean [SD], 183.8 [134.7] days vs 132.5 [124.9] days; *P* = .002) and more days of meeting their caloric intake goals than participants in the group telephone counseling group (mean [SD], 134.0 [117.2] vs 104.5 [103.0]; *P* = .049) and participants in the control group (mean [SD], 88.1 [98.3] days; *P* = .001) (Table 2).

**Table 2.** Self-Monitoring Intake Data During the 12-Month Extended Care Phase

Group	Mean (SD), d	%
Individual telephone		
Complete records	183.8 (134.7)	50.5
Caloric intake goal met	134.0 (117.2)	36.8
Group telephone		
Complete records	160.5 (130.3)	44.1
Caloric intake goal met	104.5 (103.0)	28.7
Education control		
Complete records	132.5 (124.9)	36.4
Caloric intake goal met	88.1 (98.3)	24.2

### Weight Change

**Figure 2.**
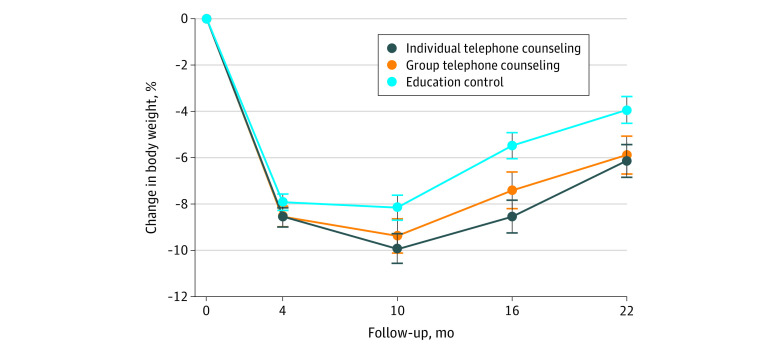
Changes in Body Weight by Group

Table 3 presents mean body weights at each assessment by group. Figure 2 displays mean percentage changes in body weight over time by group. The weight regains from months 4 to 22 were 2.3 (95% CrI, 1.2 to 3.4) kg for the individual telephone counseling group, 2.8 (95% CrI, 1.4 to 4.2) kg for the group telephone counseling group, and 4.1 (95% CrI, 3.1 to 5.0) kg for the control group. The difference in regain between the individual telephone counseling group and control group was 1.8 (95% CrI, 0.3 to 3.2) kg, and the difference between the group telephone counseling group and control groups was 1.3 (95% CrI, −0.4 to 3.0) kg. Only the individual telephone counseling vs control comparison had a 95% CrI excluding 0 (PP > .99).

**Table 3.** Weight by Month and Group

Time	Mean (95% Credible interval), kg^a^		
	Individual telephone counseling	Group telephone counseling	Education control
Baseline	98.8 (96.3-101.4)	102.0 (99.4-104.6)	98.4 (96.4-100.5)
Month			
4	90.4 (87.9-92.9)	93.3 (90.7-95.9)	90.6 (88.6-92.7)
10	89.0 (86.4-91.6)	92.5 (89.7-95.3)	90.4 (88.3-92.6)
16	90.3 (87.6-93.1)	94.5 (91.7-97.4)	93.0 (90.9-95.3)
22	92.7 (90.0-95.4)	96.1 (93.3-99.0)	94.7 (92.5-96.9)

^a^ Means were estimated based on the assumption that missing data were missing at random.

The percentage weight regains from months 4 to 22 were 2.7% (95% CrI, 1.5% to 3.8%) for the individual telephone counseling group, 2.9% (95% CrI, 1.4% to 4.4%) for the group telephone counseling group, and 4.4% (95% CrI, 3.4% to 5.5%) for the control group. The difference in percentage regain between the individual telephone counseling and control groups was 1.8% (95% CrI, 0.2% to 3.4%), and the difference between group telephone counseling and control groups was 1.5% (95% CrI, −0.3% to 3.3%). Only the individual telephone counseling vs the control comparison had a 95% CrI excluding 0 (PP > .98). Percentage changes in weight during the year-long extended care period were 0% (95% CrI, −1.1% to 1.1%) for the individual telephone counseling group, 1.3% (95% CrI, 0% to 2.5%) for the group telephone counseling group, and 2.6% (95% CrI, 1.7% to 3.6%) for the educational control group, indicating regain in the control group, a smaller but not statistically significant regain in the group telephone counseling group, and no evidence of regain in the individual telephone counseling group. During the no contact follow-up interval (months 16-22), the percentage weight changes were 2.6% (95% CrI, 1.9% to 3.3%) for the individual telephone counseling group, 1.6% (95% CrI, 0.8% to 2.4%) for the group telephone counseling group, and 1.7% (95% CrI, 1.0% to 2.4%) for the control group, indicating regains in all 3 groups.

The percentages of participants in each group who achieved body weight reductions of at least 5% at month 22 were 51.4% (95% CrI, 43.4% to 59.7%) for the individual telephone counseling group, 49.6% (95% CrI, 42.2% to 57.7%) for the group telephone counseling group, and 42.3% (95% CrI, 35.4% to 48.9%), in the control group, and the between-group differences were not statistically significant. The percentages of participants who achieved weight reductions of at least 10% at month 22 were 31.5% (95% CrI, 24.1% to 40.0%) for the individual telephone counseling group, 27.6% (95% CrI, 21.0% to 35.7 %) for the group telephone counseling group, and 19.1% (95% CrI, 14.1% to 24.9%)for the control group. The difference between individual telephone counseling and control groups in proportion of participants who achieved weight reductions of at least 10% was statistically significant (12.4% [95% CrI, 2.9% to 22.3%]; PP > .99).

The number of days that participants met their caloric intake goals partially mediated between-group differences in weight change. The average causal mediation effect for the mediator for individual telephone counseling vs control group was 0.62 (95% CrI, 0.24 to 1.10) and accounted for more than one-third of the total effect.

## Discussion

This randomized clinical trial conducted in CES offices in rural communities had 3 main findings. First, extended care delivered via individual telephone counseling produced significantly better long-term maintenance of lost weight than did an education control program. Second, a significantly larger proportion of participants who received individual telephone counseling achieved long-term weight reductions of at least 10% compared with the education control group. Third, the effect of individual telephone counseling on improved long-term maintenance of lost weight was partially mediated by greater adherence to caloric intake goals.

The benefits of individual telephone counseling demonstrated in this study replicate the findings from the Treatment of Obesity in Underserved Rural Settings trial.^19^ In that study, participants who received extended care via individual telephone counseling maintained a mean of 88% of their initial weight losses at a 12-month follow up. In our study, the participants who received individual telephone counseling maintained a mean of 100% of their initial weight losses at the 12-month follow-up. At the final follow-up, 18 months after the initial treatment, participants who received individual telephone counseling in this study sustained a mean of 72% of their initial weight losses, compared with 49% for participants in the education control group; the individual telephone counseling group had a mean net reduction in baseline body weight of 6.1%, compared with 3.9% for the control group. In addition, the proportion of individuals who achieved clinically meaningful reductions of at least 10% of body weight at 22 months was significantly greater in the individual telephone counseling group than in the control group (31% vs 19%).

Findings from the mediational analysis provide insight into a mechanism likely contributing to the enhanced maintenance observed in the individual telephone counseling intervention vs the control group. Individual telephone counseling increased both the frequency of self-monitoring and the number of days in which participants achieved their caloric intake goals compared with the control group. Ongoing contacts with a supportive counselor may set an expectation for program adherence that increases the likelihood that the participant will achieve targeted goals or engage in problem-solving efforts to overcome barriers to attaining those objectives.^36^ Providing extended care via individual telephone contacts may enhance attendance, adherence, and engagement in self-regulatory processes important to sustaining weight-loss progress.^37,38^

The group telephone counseling group in this study did not demonstrate the effectiveness observed in previous studies.^22,39^ In prior trials, the group conference call strategy has been used to induce initial weight loss and to provide extended care. In this trial, participants received face-to-face treatment during the initial weight-loss period but subsequently were assigned randomly to the group telephone counseling modality. The change in mode of treatment delivery may have contributed to the poorer performance in the group telephone counseling group in this trial compared with prior studies.^22,38^ The focused attention received by participants in the individual telephone counseling group may have minimized negative reactions to the shift from face-to-face to telephone contacts.

During the extended-care phase of this study, participants in the group telephone counseling group compared with participants in the individual telephone counseling group attended fewer sessions (54% vs 67% of sessions) and met their caloric intake goals less often (29% vs 37% of possible days). The individual telephone counseling program permitted participants to reschedule a planned session, an option not available in the group telephone counseling group. In addition, participants in the group telephone counseling group may have been reluctant to disclose their setbacks in the relatively public group setting.^40,41^ In prior trials^22,39^ in which both initial treatment and extended-care sessions were conducted entirely via group conference calls and in which the individuals had no history of face-to-face contacts with other group members, participants may have been more comfortable in discussing dietary lapses and weight gains.^42^

### Limitations

Several limitations should be noted. First, the findings were based on a study population with a community environment and sociodemographic characteristics common to adults residing in rural areas of the southeastern US; generalizability to other populations may be limited. Second, the assessment of adherence was based on uncorroborated data from daily self-monitoring records. However, a systematic review of 15 studies found a consistent and significant positive association between dietary self-monitoring and successful weight-management.^43^ Third, the benefits of individual telephone counseling must be considered in relation to its costs, particularly the time required to provide each participant with 18 individual telephone sessions. An economic analysis incorporating the perspectives of the participant, interventionist, and society may elucidate the relation of costs to benefits of extended care via individual telephone counseling.^20,44^

## Conclusions

National guidelines recommend comprehensive lifestyle intervention as the frontline treatment for people with obesity. Nonetheless, lifestyle treatment continues to be challenged by the maintenance problem and by limited data demonstrating effective dissemination into low-resource communities. The results from this randomized clinical trial inform each of these limitations. Our findings indicate that providing extended care via individual telephone counseling can enhance maintenance of lost weight and increase the proportion of participants who achieve clinically meaningful long-term reductions in body weight. The findings also demonstrate that the existing infrastructure of the CES can be mobilized for effective dissemination of comprehensive lifestyle treatment into underserved rural communities throughout the US.

## Notes

Supplement 1. **Trial Protocol**

Supplement 2. **Data Sharing Statement**
