# Supplementary material for: Effect of Telehealth Extended Care for Maintenance of Weight Loss in Rural US Communities: A Randomized Clinical Trial
Source: JAMA Netw Open. 2020 Jun 15;3(6):e206764. doi: 10.1001/jamanetworkopen.2020.6764 (PMC7296388; doi:10.1001/jamanetworkopen.2020.6764)
Supplement: Supplement 1. — Trial Protocol [file jamanetwopen-3-e206764-s001.pdf]

# Supplemental Material

## Supplement 1. Trial Protocol

# Protocol of the Rural Lifestyle Eating and Activity (Rural LEAP) Trial

**Principal Investigator:** Michael G. Perri, PhD[a]

**Co-Investigators:** Marian C. Limacher, MD[a]

David M. Janicke, PhD[a]

Kathryn M. Ross, PhD[a]

Linda B. Bobroff, PhD[a]

A. Daniel Martin, PhD[a]

Christie A. Befort, PhD[b]

**Statistician:** Michael J. Daniels, ScD[a]

**Health Economist:** Tiffany A. Radcliff, PhD[c]

**Project Coordinator:** Meena N. Shankar, MS, RD[a]

[a] University of Florida, P.O. Box 100185, Gainesville, FL 32610 USA

[b] University of Kansas Medical Center, 3901 Rainbow Blvd., Kansas City, KS 66160 USA

[c] Texas A&M University, 212 Adriance Lab Rd., College Station, TX 77843 USA

**Clinical trial number:** NCT02054624 on ClinicalTrials.gov

**Acknowledgement:** This work was supported by NHLBI grant R18 HL112720.

## Abstract

Obesity is a major contributor to the greater prevalence of chronic disease morbidity and mortality observed in rural versus nonrural areas of the U.S. Nonetheless, little research attention has been given to modifying this important driver of rural/urban disparities in health outcomes. Although lifestyle treatments produce weight reductions of sufficient magnitude to improve health, the existing research is limited with respect to the long-term maintenance of treatment effects and the dissemination of services to underserved populations. Recent studies have demonstrated the feasibility of delivering lifestyle programs through the infrastructure of the U.S. Cooperative Extension Service (CES), which has more than 2,900 offices nationwide and whose mission includes nutrition education and health promotion. In addition, several randomized trials have shown that supplementing lifestyle treatment with extended-care programs consisting of either face-to-face sessions or individual telephone counseling can improve the maintenance of weight loss. However, both options entail relatively high costs that inhibit adoption in rural communities. The delivery of extended care via group-based telephone intervention may represent a promising, cost-effective alternative that is well suited to rural residents who tend to be isolated, have heightened concerns about privacy, and report lower quality of life. The Rural Lifestyle Eating and Activity Program (Rural LEAP) is a randomized trial, conducted via CES offices in rural communities, targeted to adults with obesity (n=540), and designed to evaluate the effectiveness and cost-effectiveness of extended-care programs delivered via group or individual telephone counseling compared to an education control condition on long-term changes in body weight.

## 1. Introduction

Obesity disproportionately affects Americans living in rural areas and contributes to higher prevalences of chronic diseases and all-cause mortality observed in rural versus nonrural communities [1-6]. In rural areas, the convergent effects of poverty, low educational attainment, limited access to preventive health services, and cultural factors regarding diet and exercise contribute to higher rates of obesity and associated diseases [7-13].

Weight loss can reverse many of the adverse consequences linked to obesity [14]. According to national guidelines [15] and literature reviews [16-20], the most effective means of achieving weight reductions at the individual level is comprehensive lifestyle treatment. Participants in lifestyle interventions are taught behavioral strategies (e.g., goal setting and self-monitoring) to modify their eating and physical activity patterns so as to produce a negative energy balance and weight loss. Comprehensive lifestyle treatment routinely results in weight losses of 5-10% [16-20]. Reductions of this magnitude can produce beneficial effects on hypertension, glucose intolerance, and hyperlipidemia, and can prevent the onset of type 2 diabetes [15,21-23].

The limited availability of obesity treatment options in rural communities prevents many residents from engaging in weight-control efforts [24]. With a nationwide network of more than 2,900 offices and a mission that includes nutrition education and health promotion, the Cooperative Extension Service (CES) [25,26] has the potential to play an important role in providing treatment for obesity in rural communities.

While lifestyle interventions can produce clinically meaningful weight reductions, the long-term maintenance of those losses remains a challenge. Without follow-up care, participants commonly regain one-third to one-half of their initial weight loss within one year [18,27-31]. Providing extended care via face-to-face sessions improves long-term outcomes [28,32], but is time- and cost-intensive [29,33,34], thereby limiting adoption in low-resources

settings. Delivering extended care via telephone counseling may decrease the burdens associated with face-to-face treatment while producing equivalent weight-loss outcomes [35].

Most studies supporting phone-based interventions (e.g., [31,35]) have utilized *individual* telephone counseling, which requires a significant amount of provider time and consequently poses a major barrier to widespread implementation. Group-based phone counseling may overcome this challenge, but evidence of its effectiveness is limited. Befort and colleagues [36] found that group phone counseling produced weight losses equivalent to individual phone counseling, but at lower costs, and in a subsequent trial [37] showed that extended care delivered via group phone counseling improved weight-loss maintenance compared with a newsletter control group. In an efficacy study conducted largely with urban participants, Donnelly et al. [38] found equivalent outcomes for lifestyle treatment delivered via face-to-face sessions versus group conference call counseling. However, the impact of extended-care interventions delivered via group- versus individual telephone counseling in less resourced, rural areas remains unknown.

The Rural Lifestyle Eating and Activity Program (Rural LEAP) is a randomized trial designed to evaluate the comparative effectiveness and cost efficiency of three methods of delivering extended-care counseling for weight-loss maintenance in rural communities. The intervention will be delivered through the infrastructure of the CES, and the primary outcome is weight change over the course of 18 months following initial treatment.

## 2. Methods

### *2.1. Overview*

The study will consist of three distinct phases: (1) Phase I, a non-randomized 4-month weight-loss phase (Months 1 through 4) during which all participants receive the same face-to-face group lifestyle intervention for weight loss; (2) Phase II, a 12-month extended-care phase (Months 5 through 16) during which participants receive additional treatment according to randomized assignment to group-based (GRP) phone counseling, individual-based (IND) phone

counseling, or an education control (CTRL) group; and (3) Phase III, a 6-month no-contact follow-up period (Months 17 through 22).

### *2.2. Participants*

The study was approved by the Institutional Review Board of the University of Florida. Participants will include 540 adults, 21-75 years of age, with body mass indices (BMI) of 30-45 $kg/m^2$. Eligible participants will be free of uncontrolled diabetes and hypertension and had no active manifestations of cardiovascular, cerebrovascular, renal, or hepatic disease. Exclusion criteria included the use of medications known to affect body weight, musculoskeletal conditions that precluded walking for 30 minutes, and weight loss > 4.5 kg in the preceding 6 months. Psychosocial contraindications included clinically significant depression and substance abuse.

### *2.3. Recruitment and Screening*

Study announcements will be mailed to residential addresses within 14 rural counties in northern Florida [39]. All 14 counties are designated in whole or in part as "Health Professional Shortage Areas" [40]. In response to the mailings and other outreach activities (e.g., presentations at churches and community events), interested adults will complete a preliminary telephone screening to determine if they meet basic eligibility criteria for study participation. An in-person screening visit will then then conducted at a local CES site during which the study will be described and informed consent obtained. At the screening visit, a medical history will be collected, and a study nurse will measure the potential participant's height, weight, and resting blood pressure, and a fasting blood sample will be drawn and analyzed for metabolic and lipid profiles. Following review of the screening visit results, individuals who meet the eligibility criteria will be enrolled into Phase I of the study.

### *2.4. CES Offices and Interventionists*

The initial weight-loss intervention will be delivered on site via CES sites in 14 rural counties in northern Florida. All 14 counties have a centrally-located site available for the intervention. Interventionists are CES Family and Consumer Sciences Agents or individuals

with a bachelor's or master's degree in nutrition, exercise science, or psychology. All interventionists will be provided with training in lifestyle treatment and nutrition education that includes two 8-hour workshops held semi-annually, plus case management reviews with a clinical health psychologist (MGP) or registered dietitian (MNS), each with extensive experience overseeing behavioral interventions for obesity. The case management reviews are conducted weekly during Months 1 through 4, biweekly during Months 5 through 10, and monthly during Months 11 through 16. Interventions sessions are audio-taped and random reviews are carried out to ensure treatment fidelity. Corrective feedback is provided during training, case management reviews, or individually with the interventionists.

### *2.5. Phase I Lifestyle Intervention*

Phase I will consist of 16 weekly face-to-face group sessions conducted at local CES offices. Each group will consist of 4-16 participants, and each session will last approximately 90 minutes. Intervention content was derived from the Diabetes Prevention Program (DPP) [42,43]. Modifications to the DPP approach include group rather than individual counseling [44] and home-based rather than center-based physical activity [45]. The DPP intervention was further modified to address weight-management challenges experienced by individuals living in rural areas (i.e., lack of places to exercise, traditions of high-fat Southern cooking, absence of social support for weight loss, etc.). Similar to the DPP, the Rural LEAP intervention is guided by social-cognitive and self-regulation theories [46-48], and it incorporates key behavioral modification strategies including goal setting, self-monitoring of dietary intake and physical activity, stimulus control, cognitive restructuring, and problem solving [33]. Utilization of problem-solving skills, in particular, is emphasized as a way to address the specific challenges experienced by adults living in rural communities [49,50]. Topics and objectives for the 16 Phase I and 18 Phase II weight-loss treatment modules are presented in Table 2.

The goals of the initial lifestyle intervention are to decrease caloric intake in a nutritionally sound manner [51-53] so as to produce a weight loss of approximately 0.4-0.9 kg

per week and to increase home-based walking to 210 minutes per week [54]. Initial caloric intake goals are determined by the participant's baseline weight (e.g., 1200 kcal/day for participants weighing ≤ 113.6 kg; 1500 kcal/day for those weighing > 113.6 kg). Participants will be instructed to keep daily logs of their food and drink intake, including the types and amounts of foods consumed along with corresponding caloric values, using either written logs or online programs/applications (e.g., MyFitnessPal® or Lose It!®). Participants will also be instructed to keep daily records of their steps using a pedometer or activity monitor. To support these self-monitoring efforts, participants will be provided, at no cost, with a *Calorie King* reference book [55,56], blank food logs, measuring cups, a food scale, and a clip-on pedometer. As a substitute for the use of a pedometer, participants will be permitted to self-monitor physical activity via self-purchased electronic tracking devices (e.g., Fitbit®, Garmin®, or Apple Watch®).

Participants will be encouraged to work toward meeting their daily caloric intake goals by selecting lower-calorie food options, decreasing consumption of "fast food," decreasing or eliminating sugar-sweetened beverages, increasing consumption of fruits, vegetables, and whole grains, and using lower-fat methods of preparing foods. Weight-loss treatment modules included instruction on the USDA's *MyPlate* to emphasize choosing whole grains, fruits, vegetables, lean meat, and low-fat dairy products [51].

Walking will be the primary form of prescribed physical activity. During week 2, each participant's steps will be monitored over 7 days, and the participants will then be encouraged to gradually increase their planned daily walking time by 5-10 minutes (i.e., 500-1000 steps) each week as tolerated until they reach an average of 30 minutes/day (3000 steps) above their baseline level. Participants who achieve this objective will be encouraged to further increase their steps toward an ultimate goal of 60 min/day of walking for exercise (i.e., 6000 steps above baseline).

### ***2.6. Phase II Randomization and Extended-Care Lifestyle Intervention***

To maximize participation in the randomized and follow-up phases (II and III) of the trial, only those individuals who attend fewer than 50% of the Phase I sessions will be excluded from advancing to Phase II randomized assignments. All other participants, regardless of initial weight loss achieved, will be randomly assigned to one of the three Phase II conditions (GRP, IND, or CTRL). Randomization was completed by the study statistician (MJD) using the random number generator in R statistical computing software [57]. County size, county, Phase I group, and session time will be balanced during randomization. Interventionists will notify participants of their Phase II group assignment during the last Phase I session. The primary objectives of Phase II (Months 5-16) are to sustain the lifestyle changes accomplished in Phase I so as to maintain lost weight and to make further adjustments in energy balance to produce additional weight loss during “campaigns” conducted in Months 8-9 and Months 13-14.

The mode of extended-care contacts will differ by condition during Phase II, but the schedule of contacts is consistent across the conditions. Intervention contact across all three conditions will occur bi-weekly during Month 5 through 10 and monthly during Months 11 through 16. At each of the scheduled contact points, participants in all three conditions receive a weight-loss treatment module delivered via e-mail or U.S. mail with information and recommended behavioral activities targeted at maintaining lost weight. All participants will also be provided with a supply of blank self-monitoring logs and pre-paid mailers for returning completed dietary logs to the interventionists.

***2.6.1. GRP condition***

Participants randomized to the GRP condition will call into a teleconference line for 60-minute group sessions with the interventionist and other members from their Phase I group. The calls will include three primary components: (1) a group “check-in” during which participants report progress toward goals and problems experienced since the previous session coupled with group problem solving of one or two complex issues to address barriers to progress [49]; (2) group discussion of the treatment module with the interventionist explaining new objectives

and suggesting behavioral strategies to achieve those objectives; and (3) structured individual goal setting with respect to each participant's plans for diet and/or physical activity changes to be accomplished prior to the next session. To maximize the time efficiency of the group session, all participants will be asked to identify their calorie and step goals *prior* to the call.

The group problem-solving activities facilitated by the interventionist during the extended-care sessions will incorporate the use of an evidence-based, five-step model [49,50] including: (1) orientation (i.e., developing an appropriate coping perspective -- “Problems are a normal part of managing your weight, but they can be dealt with effectively.”); (2) definition (i.e., specifying the problem and goal behaviors – “What is the particular problem facing you right now? What is your goal in this situation?”); (3) generation of alternatives (i.e., brainstorming potential solutions – “The greater the range of possible solutions you consider, the greater your chances of developing an effective solution.”); (4) decision making (i.e., anticipating the probable outcomes of different options – “What are the likely short- and long-term consequences of each of your options?”; and (5) implementation and evaluation (i.e., trying out a plan and evaluating its effectiveness – “What solution plan are you going to try and how will you know if it works?”).

***2.6.2. IND condition***

Participants randomized to the IND condition call into a teleconference line at a predetermined appointment time for a one-on-one, 10- to 20-minute session with the interventionist. Participants will be contacted to schedule make-up calls in the event of time conflicts or missed calls. Aside from the difference in call length and the absence of other group members, IND calls will include the same structure, components, and problem-solving focus of the GRP calls.

***2.6.3. CTRL condition***

Participants in the CTRL group will receive a series of 18 written educational weight-loss treatment modules delivered via e-mail and/or U.S. mail delivered on the same schedule as the

GRP and IND contacts during Phase II. The modules will include the identical content used in the GRP and IND conditions. The CTRL condition will not include any scheduled phone or in-person interactions with the interventionists or other participants.

### *2.7. Measures*

#### *2.7.1. Assessment visits*

Study personnel masked to participant randomization will conduct in-person assessment visits at baseline and at Months 4, 10, 16, and 22. Assessment visits will be conducted at the same local CES site for all time points. Self-reported medical history and a list of medications will be updated at each assessment visit.

#### *2.7.2. Anthropometrics*

At baseline, height will be measured using a stadiometer (ShorrBoard®), with participant's shoes removed. At baseline and subsequent assessment visits, weight will be measured with a calibrated digital scale (Tanita BWB-800S), with participants in light indoor clothing, pockets emptied, and shoes removed.

#### *2.7.3. Fasting blood samples*

Fasting blood samples will be obtained by the study nurse at baseline and at Months 4 and 22 and analyzed for metabolic and lipid profiles by Quest Diagnostics®.

#### *2.7.4. Resting blood pressure and heart rate*

Blood pressure and heart rate will be measured at baseline and Months 4 and 22. Measurements will be taken while the participant is seated with feet resting on the floor for five minutes in a quiet room free of distractions. Resting systolic and diastolic blood pressure will be taken three times, two minutes apart, using a Dinamap® Automated Vital Signs Monitor with appropriate cuff size; the second and third readings will be entered and averaged in the study database.

#### *2.7.5. 400 Meter Walk Test*

The 400 Meter Walk Test [58], a standardized measure of physical fitness commonly used to assess individuals with chronic health conditions, will be completed at baseline, and at Months 4 and 22 to assess fitness.

### *2.7.6. Participant questionnaires*

The following questionnaires will be completed by the participants at baseline and at Months 4, 10, 16, and 22.

#### *2.7.6.1. Paffenbarger Physical Activity Questionnaire*

Self-reported physical activity will be collected via the Paffenbarger Physical Activity Questionnaire [59], which includes questions on distance walked, flights of stairs climbed, and time spent in other sports, recreational, or fitness activities during a typical day or week.

#### *2.7.6.2. Medical Outcomes Study 36-Item Short Form Health Survey (SF-36)*

The SF-36 [60] is a quality-of-life measure that assesses the following domains: (a) physical functioning; (b) role limitations due to physical health; (c) bodily pain; (d) general health; (e) vitality; (f) social functioning; (g) role limitations due to emotional problems; and (h) mental health [61].

#### *2.7.6.3. Social Problem-Solving Inventory-Revised (SPSI-R)*

The SPSI-R [62-64] is a 52-item questionnaire that evaluates positive versus negative problem orientation and problem-solving style.

#### *2.7.6.4. Social Provisions Scale*

The 24-item Social Provisions Scale [65,66] measures perceived support from others in one’s social network.

#### *2.7.6.5. Cost Analysis Questionnaire*

The cost analysis questionnaire [35] assesses direct and indirect costs of lifestyle interventions.

#### *2.7.6.6. Health Thermometer*

The Health Thermometer is a visual analog scale [67] that measures health-related quality of life on a scale of 0 to 100 with 0 indicating the “worst imaginable health state” to 100 indicating the “best imaginable health state.”

***2.7.6.7. Program Satisfaction and Group Leader Evaluation Forms***

Participant ratings of program satisfaction, the usefulness of specific treatment strategies (e.g., self-monitoring, cognitive restructuring, etc.), and the effectiveness of interventionists are completed at Months 4, 10, 16, and 22.

***2.8. Participant Retention Strategies***

Participants will receive payments on a study-provided credit card of $50 upon completion of the Month 4 visit, $20 upon the completion of the Month 10 and Month 16 visits, and $75 upon completion of the Month 22 visit.  Participants will also receive $10 as travel reimbursement for each of the in-person sessions attended during Phase I.

***2.9. Data Management and Statistical Analyses***

***2.9.1. Data management***

Data will be stored in a database via the Research Electronic Data Capture (REDCap) system [68], a secure web-based data management system supported by the University of Florida Clinical and Translational Science Institute.  Self-report data will be entered directly by participants into REDCap’s online survey module.  All other data will be entered by study personnel according to the protocol and in compliance with the U.S. Health Insurance Portability and Accountability (HIPAA) Privacy Rule.  Data will be exported to a SAS dataset for statistical analyses [69].

***2.9.2. Missing data***

Data analyses for this effectiveness trial will be performed using an intent-to-treat approach that includes all randomized participants.  It will be assumed that individuals who discontinue participation in the study prior to Month 22 regained weight, on average, at a rate of 0.3 kg per month after leaving the study, up to their baseline weight.  This conservative

approach to imputing missing weight values has been employed by numerous prior studies [34,35,70] and is consistent with published reviews of weight regain following lifestyle treatment for obesity [18,71,72]. We will also consider missing at random. Sensitivity to the assumptions about the missing data [75] will be examined in our final analysis.

***2.9.3. Sample size justification***

The primary outcome will be change in body weight from Month 4 to Month 22, comparing the GRP and IND phone-based conditions to the education CTRL condition. It is assumed that the county size, county, and session time effects will be negligible due to the balance in the randomization assignments. The analysis will, however, take into account a random effect that allows for correlation among individuals in the same GRP sessions. Based upon prior studies [35,76], a 20% attrition rate is expected by Month 22. The recruitment target includes a total of 540 individuals entering Phase I (180 per condition). Power calculations were computed using the expected attrition rate and a Bonferroni adjustment to account for comparisons of the three treatment conditions. The study is expected to have > 80% power (two-sided tests, type I error rate of 0.05) to detect a 2.5 kg difference in regain from Month 4 to Month 22 between GRP vs. CTRL and IND vs. CTRL.

***2.9.4. Primary analyses***

Participants' weight change will be calculated as the difference between weight in kilograms at Month 22 and weight at Month 4. The primary hypothesis is that both the GRP and IND telephone conditions will produce greater weight reductions at Month 22 than the education CTRL condition. To test this hypothesis, we will employ the NiNBayes R package (github.com/theodds/NiNBayes), which models the weights longitudinally using a Dirichlet process mixture of models.[77] Treatment condition is randomly assigned. Two primary contrasts of interest will be tested for significance: GRP vs. CTRL and IND vs. CTRL. Bonferroni corrections will be used to control for type I error. In addition, percent change in body

weight during months 4 to 22 will be analyzed by computing percent changes in body weight from the models used for the primary outcome, weight change.

***2.9.5. Secondary analyses***

For the secondary aim, the outcomes of interest are the proportions of participants within a treatment condition who lost ≥ 5% and ≥ 10% of body weight at Month 22. It is hypothesized that both the GRP and IND conditions will result in greater proportions of participants with ≥ 5% and ≥10% losses at Month 22 than the CTRL condition. Two contrasts will be tested for significance: GRP vs. CTRL and IND vs. CTRL. Based on the Bayesian approach for the primary outcome and missingness, for each contrast, we will test the null hypothesis that the relevant proportions are equal versus the alternative that they are not equal.

***2.9.6. Additional analyses***

***2.9.6.1. Exploratory analyses.*** Changes from Month 4 to Month 22 will be examined for systolic and diastolic blood pressure, resting heart rate, blood lipids profile (LDL and HDL cholesterol and triglycerides), glycemic control (HbA1c), dietary intake, self-reported physical activity, physical performance (400 Meter Walk Test [58]), and health-related quality of life. Process measures will include intervention-related activities (e.g., attendance, self-monitoring) and participants' evaluation of program components and interventionist effectiveness. These endpoints are continuous variables, and analyses will be the same as that described for the primary endpoint. No adjustments will be made for multiple comparisons due to the exploratory nature of these analyses.

***2.9.6.2. Mediator analysis.*** Adherence to caloric intake goals will be evaluated as a mediator of the relationship between the intervention conditions and weight change at Month 22. Causal mediation analyses will be conducted using the mediate function from the mediation R package [78].

***2.9.6.4. Cost-effectiveness analyses.*** The analytic framework will follow the guidelines of the Second Panel on Cost-Effectiveness in Health and Medicine [79]. Costs of each condition

will be tracked from the service provider perspective [35]. Cost effectiveness will be measured primarily as “dollars per kg loss per treatment condition” [80]. Cost effectiveness will also be measured according to the recommended clinical cut-points of maintaining ≥ 5% loss of initial weight [15]. Similar effectiveness for the GRP and IND conditions are expected, with lower effectiveness in the CTRL condition. Cost effectiveness will also be assessed based on Quality-Adjusted Life Year (QALY) gains related to weight change and will be calculated using data from the SF-36 questionnaires completed at baseline, Month 4, Month 16, and Month 22 to derive a preference score using the Short Form-6 Dimension (SF6D) method; 10 items from the SF-36 are used to calculate a QALY score [81].

Incremental cost effectiveness ratios (ICERs) will identify the differential costs and outcomes of switching from individual to group-based telephone counseling. A priori, it is expected that the GRP condition will be less expensive than the IND condition but with similar effectiveness. All costs will be adjusted to reflect constant 2017 dollars. Sensitivity analyses will be conducted to determine whether costs and cost-effectiveness estimates are sensitive to study-specific wage rates and other program costs. Extrapolation using sample median wage rates or national wage data will be calculated and the analysis will be rerun to determine any change in results. Additional sensitivity analyses will rely on either probabilistic models or tornado charts to gauge whether ICER results depend on various model assumptions.

## References


1. Befort CA, Nazir N, Perri MG. Prevalence of obesity among adults from rural and urban areas of the United States: Findings from NHANES (2005-2008). J Rural Health. 2012;28(4):392-7. https://doi.org/10.1111/j.1748-0361.2012.00411.x
2. Lundeen EA, Park S, Pan L, O'Toole T, Matthews K, Blanck HM. Obesity prevalence among adults living in metropolitan and nonmetropolitan counties-United States, 2016. MMWR Morb Mortal Wkly Rep. 2018;67(23):653-58. https://doi.org/10.15585/mmwr.mm6723a1
3. Hales CM, Fryar CD, Carroll MD, Freedman DS, Aoki Y, Ogden CL. Differences in obesity prevalence by demographic characteristics and urbanization level among adults in the United States, 2013-2016. JAMA. 2018;319(23):2419-29. https://doi.org/10.1001/jama.2018.7270
4. Wen M, Fan JX, Kowaleski-Jones L, Wan N. Rural-urban disparities in obesity prevalence among working age adults in the United States: Exploring the mechanisms. Am J Health Promot. 2018;32(2):400-08. https://doi.org/10.1177/0890117116689488
5. Knudson A, Meit, M., Tanenbaum, E., Brady, J., Gilbert, T., Klug, et al. 2016, March; Available from: https://ruralhealth.und.edu/projects/health-reform-policy-research-center/pdf/technical-notes-exploring-rural-urban-mortality-differences.pdf.
6. Moy E. Leading causes of death in nonmetropolitan and metropolitan areas-United States, 1999–2014. MMWR Surveillance Summary; 2017; 66: Available from: http://www.cdc.gov/mmwr/volumes/66/ss/ss6601a1.htm.
7. Jones CP, TS; Ahearn, M.; et al. Health status and healthcare access of farm and rural populations. United States Department of Agriculture Economic Research Service, 2009.
8. Martin SL, Kirkner GJ, Mayo K, Matthews CE, Durstine JL, Hebert JR. Urban, rural, and regional variations in physical activity. J Rural Health. 2005;21(3):239-44.
9. Patterson PD, Moore CG, Probst JC, Shinogle JA. Obesity and physical inactivity in rural America. J Rural Health. 2004;20(2):151-9.
10. Rural Healthy People 2020: A Companion Document to Healthy People 2020, volume 1. Texas A&M University Health Science Center, School of Public Health, Southwest Rural Health Research Center, 2015.
11. Flora CB, Flora, JL, & Gasteyer, SP. Rural communities: Legacy + change. Boulder, Colorado: Westview Press; 2015.
12. Lutfiyya MN, Chang LF, Lipsky MS. A cross-sectional study of US rural adults' consumption of fruits and vegetables: Do they consume at least five servings daily? BMC Public Health. 2012;12:280. https://doi.org/10.1186/1471-2458-12-280
13. Pearson TA, Lewis C. Rural epidemiology: Insights from a rural population laboratory. Am J Epidemiol. 1998;148(10):949-57.
14. McTigue KM, Harris R, Hemphill B, Lux L, Sutton S, Bunton AJ, et al. Screening and interventions for obesity in adults: Summary of the evidence for the U.S. Preventive Services Task Force. Ann Intern Med. 2003;139(11):933-49.
15. Jensen MD, Ryan DH, Apovian CM, Ard JD, Comuzzie AG, Donato KA, et al. 2013 AHA/ACC/TOS guideline for the management of overweight and obesity in adults: A report of the American College of Cardiology/American Heart Association Task Force on Practice Guidelines and The Obesity Society. Circulation. 2014;129(25 Suppl 2):S102-38. https://doi.org/10.1161/01.cir.0000437739.71477.ee
16. Butryn ML, Webb V, Wadden TA. Behavioral treatment of obesity. Psychiatr Clin North Am. 2011;34(4):841-59. https://doi.org/10.1016/j.psc.2011.08.006
17. Wing RR. Behavioral weight control. Handbook of obesity treatment. New York, NY: Guilford Press; 2002: 301-316.

18. Wadden TA, Butryn ML, Wilson C. Lifestyle modification for the management of obesity. Gastroenterology. 2007;132(6):2226-38. https://doi.org/10.1053/j.gastro.2007.03.051
19. Johnston CA, Tyler C, Foreyt JP. Behavioral management of obesity. Curr Atheroscler Rep. 2007;9(6):448-53.
20. Vetter ML, Faulconbridge LF, Webb VL, Wadden TA. Behavioral and pharmacologic therapies for obesity. Nat Rev Endocrinol. 2010;6(10):578-88. https://doi.org/10.1038/nrendo.2010.121
21. Chen L, Pei JH, Kuang J, Chen HM, Chen Z, Li ZW, et al. Effect of lifestyle intervention in patients with type 2 diabetes: A meta-analysis. Metabolism. 2015;64(2):338-47. https://doi.org/10.1016/j.metabol.2014.10.018
22. Lin JS, O'Connor E, Evans CV, Senger CA, Rowland MG, Groom HC. Behavioral counseling to promote a healthy lifestyle in persons with cardiovascular risk factors: A systematic review for the U.S. Preventive Services Task Force. Ann Intern Med. 2014;161(8):568-78. https://doi.org/10.7326/M14-0130
23. Knowler WC, Barrett-Connor E, Fowler SE, Hamman RF, Lachin JM, Walker EA, et al. Reduction in the incidence of type 2 diabetes with lifestyle intervention or metformin. N Engl J Med. 2002;346(6):393-403. https://doi.org/10.1056/NEJMoa012512
24. van Dis J. Where we live: Health care in rural vs urban America. JAMA. 2002;287(1):108-08. https://doi.org/DOI 10.1001/jama.287.1.108
25. United States Department of Agriculture. Cooperative Extension System, National Institute of Food and Agriculture. 2015; Available from: https://nifa.usda.gov/cooperative-extension-system.
26. Braun B BK, Cronk L, Fox LK, Koukel S, Le Menestrel S et al. Cooperative Extension's National Framework for Health and Wellness. 2014; Available from: http://www.aplu.org/members/commissions/food-environmentand-renewable-resources/CFERR_Library/national-framework-for-health-and-wellness/file.
27. Appel LJ, Clark JM, Yeh HC, Wang NY, Coughlin JW, Daumit G, et al. Comparative effectiveness of weight-loss interventions in clinical practice. N Engl J Med. 2011;365(21):1959-68. https://doi.org/10.1056/NEJMoa1108660
28. Bray GA, Wadden TA. Improving long-term weight loss maintenance: Can we do it? Obesity (Silver Spring). 2015;23(1):2-3. https://doi.org/10.1002/oby.20964
29. Jeffery RW, Drewnowski A, Epstein LH, Stunkard AJ, Wilson GT, Wing RR, et al. Long-term maintenance of weight loss: Current status. Health Psychol. 2000;19(1S):5-16.
30. Look AHEAD Research Group. Long-term effects of a lifestyle intervention on weight and cardiovascular risk factors in individuals with type 2 diabetes mellitus: Four-year results of the Look AHEAD trial. Arch Intern Med. 2010;170(17):1566-75. https://doi.org/10.1001/archinternmed.2010.334
31. Svetkey LP, Stevens VJ, Brantley PJ, Appel LJ, Hollis JF, Loria CM, et al. Comparison of strategies for sustaining weight loss: The weight loss maintenance randomized controlled trial. JAMA. 2008;299(10):1139-48. https://doi.org/10.1001/jama.299.10.1139
32. Franz MJ, VanWormer JJ, Crain AL, Boucher JL, Histon T, Caplan W, et al. Weight-loss outcomes: A systematic review and meta-analysis of weight-loss clinical trials with a minimum 1-year follow-up. J Am Diet Assoc. 2007;107(10):1755-67. https://doi.org/10.1016/j.jada.2007.07.017
33. Look AHEAD Research Group. The Look AHEAD study: A description of the lifestyle intervention and the evidence supporting it. Obesity (Silver Spring). 2006;14(5):737-52. https://doi.org/10.1038/oby.2006.84
34. Wing RR, Tate DF, Gorin AA, Raynor HA, Fava JL. A self-regulation program for maintenance of weight loss. N Engl J Med. 2006;355(15):1563-71. https://doi.org/10.1056/NEJMoa061883

35. Perri MG, Limacher MC, Durning PE, Janicke DM, Lutes LD, Bobroff LB, et al. Extended-care programs for weight management in rural communities: The Treatment of Obesity in Underserved Rural Settings (TOURS) randomized trial. Arch Intern Med. 2008;168(21):2347-54. https://doi.org/10.1001/archinte.168.21.2347
36. Befort CA, Donnelly JE, Sullivan DK, Ellerbeck EF, Perri MG. Group versus individual phone-based obesity treatment for rural women. Eat Behav. 2010;11(1):11-7. https://doi.org/10.1016/j.eatbeh.2009.08.002
37. Befort CA, Klemp JR, Sullivan DK, Shireman T, Diaz FJ, Schmitz K, et al. Weight loss maintenance strategies among rural breast cancer survivors: The rural women connecting for better health trial. Obesity (Silver Spring). 2016;24(10):2070-7. https://doi.org/10.1002/oby.21625
38. Donnelly JE, Goetz J, Gibson C, Sullivan DK, Lee R, Smith BK, et al. Equivalent weight loss for weight management programs delivered by phone and clinic. Obesity (Silver Spring). 2013;21(10):1951-9. https://doi.org/10.1002/oby.20334
39. United States Census Bureau. Geography: Urban and rural. 2016; Available from: https://www.census.gov/geo/reference/urban-rural.html
40. United States Health and Human Services. Health professional shortage areas of primary care, dental care, or mental health providers. 2018; Available from: http://hpsafind.hrsa.gov/HPSASearch.
41. Obesity: Preventing and managing the global epidemic. Report of a WHO consultation. World Health Organ Tech Rep Ser. 2000;894:i-xii, 1-253.
42. Diabetes Prevention Program Research Group. Design and methods for a clinical trial in the prevention of type 2 diabetes. Diabetes Care. 1999;22(4):623-34.
43. Diabetes Prevention Program Research Group. The Diabetes Prevention Program (DPP): Description of lifestyle intervention. Diabetes Care. 2002;25(12):2165-71.
44. Renjilian DA, Perri MG, Nezu AM, McKelvey WF, Shermer RL, Anton SD. Individual versus group therapy for obesity: Effects of matching participants to their treatment preferences. J Consult Clin Psychol. 2001;69(4):717-21.
45. Perri MG, Martin AD, Leermakers EA, Sears SF, Notelovitz M. Effects of group- versus home-based exercise in the treatment of obesity. J Consult Clin Psychol. 1997;65(2):278-85.
46. Bandura A. Social Foundations of Thought and Action: A Social Cognitive Theory. Englewood Cliffs, NJ: Prentice-Hall; 1986.
47. Bandura A. Self-efficacy: The Exercise of Control. New York, NY: W.H. Freeman and Company; 1997.
48. Kanfer, FH, Gaelick-Buys, L. Self-management methods. Pergamon general psychology series, Vol. 52. Helping people change: A textbook of methods. Elmsford, NY: Pergamon Press, 1991: 305-360.
49. Perri MG, Nezu AM, McKelvey WF, Shermer RL, Renjilian DA, Viegener BJ. Relapse prevention training and problem-solving therapy in the long-term management of obesity. J Consult Clin Psychol. 2001;69(4):722-6.
50. Perri MG, Nezu AM, Viegener BJ. Improving the long-term management of obesity: Theory, research, and clinical guidelines. New York: Wiley; 1992.
51. United States Department of Agriculture. Choose MyPlate. 2018; Available from: https://www.choosemyplate.gov
52. United States Department of Health and Human Services, Department of Agriculture, Dietary Guidelines Advisory Committee. Dietary Guidelines for Americans, 2010. Seventh edition. Washington, D.C.
53. United States Department of Health and Human Services, Department of Agriculture, Dietary Guidelines Advisory Committee. Dietary Guidelines for Americans, 2015-2020. Eighth edition. Washington, D.C.

54. Donnelly JE, Blair SN, Jakicic JM, Manore MM, Rankin JW, Smith BK, et al. American College of Sports Medicine Position Stand. Appropriate physical activity intervention strategies for weight loss and prevention of weight regain for adults. Med Sci Sports Exerc. 2009;41(2):459-71. https://doi.org/10.1249/MSS.0b013e3181949333
55. Borushek A. The Calorie King Calorie, Fat, & Carbohydrate Counter. 2014 ed. Costa Mesa, CA: Family Health Publications; 2014.
56. Borushek A. The Calorie King Calorie, Fat, & Carbohydrate Counter. 2017 ed. Costa Mesa, CA: Family Health Publications; 2017.
57. R Core Team. R: A language and environment for statistical computing. R Foundation for Statistical Computing, Vienna, Austria. 2017.
58. Simonsick EM, Montgomery PS, Newman AB, Bauer DC, Harris T. Measuring fitness in healthy older adults: The Health ABC Long Distance Corridor Walk. J Am Geriatr Soc. 2001;49(11):1544-8.
59. Paffenbarger RS, Jr., Blair SN, Lee IM, Hyde RT. Measurement of physical activity to assess health effects in free-living populations. Med Sci Sports Exerc. 1993;25(1):60-70.
60. Jenkinson C, Wright L, Coulter A. Criterion validity and reliability of the SF-36 in a population sample. Qual Life Res. 1994;3(1):7-12.
61. American Psychological Association. Medical Outcomes Scale. n.d.; Available from: http://www.apa.org/pi/about/publications/caregivers/practice-settings/assessment/tools/medical-outcomes.aspx.
62. Maydeu-Olivares A, & Dzurilla, T. J. A factor-analytic study of the Social Problem-Solving Inventory: An integration of theory and data. Cognitive Therapy and Research. 1996;20(2):115-33.
63. Shewchuk RM, Johnson MO, Elliott TR. Self-appraised social problem solving abilities, emotional reactions and actual problem solving performance. Behav Res Ther. 2000;38(7):727-40.
64. D'Zurilla TJ NA, Maydeu-Olivares A. Social Problem-Solving Inventory-Revised: Technical manual. North Tonawanda, NY: Multi-Health Systems; 2002.
65. Cutrona CE. Objective determinants of perceived social support. J Pers Soc Psychol. 1986;50(2):349-55.
66. Cutrona CE, & Russell, D. W. The provisions of social relationships and adaptation to stress. Greenwich, CT: JAI Press; 1987.
67. Llach XB, Herdman M, Schiaffino A. Determining correspondence between scores on the EQ-5D "thermometer" and a 5-point categorical rating scale. Med Care. 1999;37(7):671-77. https://doi.org/Doi 10.1097/00005650-199907000-00007
68. Harris PA, Taylor R, Thielke R, Payne J, Gonzalez N, Conde JG. Research electronic data capture (REDCap)--a metadata-driven methodology and workflow process for providing translational research informatics support. J Biomed Inform. 2009;42(2):377-81. https://doi.org/10.1016/j.jbi.2008.08.010
69. SAS Institute Inc. SAS/ACCESS® 9.4 Interface to ADABAS. Cary, NC: SAS Institute Inc.; 2013.
70. Wadden TA, Berkowitz RI, Womble LG, Sarwer DB, Phelan S, Cato RK, et al. Randomized trial of lifestyle modification and pharmacotherapy for obesity. N Engl J Med. 2005;353(20):2111-20. https://doi.org/10.1056/NEJMoa050156
71. Olson K, Bond D, Wing RR. Behavioral approaches to the treatment of obesity. R I Med J. 2017;100(2):21-24.
72. Perri MG, Corsica JA. Improving the maintenance of weight lost in behavioral treatment of obesity. Handbook of Obesity Treatment. 2002 Jan 9;1:357-79.
73. Daniels MJ, Hogan JW. Reparameterizing the pattern mixture model for sensitivity analyses under informative dropout. Biometrics. 2000;56(4):1241-8.

74. Little RJ. A class of pattern-mixture models for normal incomplete data. Biometrika. 1994;81(3):471-83.
75. Daniels MJ, Hogan JW. Missing data in longitudinal studies: Strategies for bayesian modeling and sensitivity analysis. Boca Raton: Chapman & Hall/CRC; 2008.
76. Perri MG, Limacher MC, von Castel-Roberts K, Daniels MJ, Durning PE, Janicke DM, et al. Comparative effectiveness of three doses of weight-loss counseling: Two-year findings from the Rural LITE trial. Obesity (Silver Spring). 2014;22(11):2293-300. https://doi.org/10.1002/oby.20832
77. Daniels MJ, Hogan JW. *Missing Data in Longitudinal Studies: Strategies for Bayesian Modeling and Sensitivity Analysis.* Chapman and Hall/CRC; 2008.
78. Imai K, Keele L, Yamamoto T. Identification, inference, and sensitivity analysis for causal mediation effects. *Statistical Science* 2010;25(1):51-71. http://www.jstor.org/stable/41058997.
79. Sanders GD, Neumann PJ, Basu A, Brock DW, Feeny D, Krahn M, et al. Recommendations for conduct, methodological practices, and reporting of cost-effectiveness analyses: Second panel on cost-effectiveness in health and medicine. JAMA. 2016;316(10):1093-103. https://doi.org/10.1001/jama.2016.12195
80. Institute of Medicine. Weighing the Options: Criteria for Evaluating Weight-Management Programs. Thomas PR, ed. Washington, DC: National Academies Press; 1995.
81. Brazier J, Roberts J, Deverill M. The estimation of a preference-based measure of health from the SF-36. J Health Econ. 2002;21(2):271-92.

**Table 1**

Weight-loss treatment modules.

| Session | Topic | Objective |
|---|---|---|
| 1 | Getting Started for Success: Setting Goals | Review energy balance and weight management. Set personal calorie intake goals. |
| 2 | Taking Control of Eating Patterns | Learn difference between hunger vs. cravings. Review strategies to implement healthy eating patterns. |
| 3 | Stepping Up: Becoming Active | Review benefits of physical activity. Set personal step goals. |
| 4 | Becoming a Fat Gram Detective/Southern Cooking Made Healthier | Understand roles of fat, traditional Southern foods, and calories in long-term weight management. Review lower-fat food options. |
| 5 | Building a Healthy Diet | Review MyPlate recommended servings for each food group. Set total daily food serving goals. |
| 6 | Keep on Moving! | Discuss benefits of exercise/aerobic fitness and safety. |
| 7 | Scheduling Sleep | Identify current sleep habits and learn how sleep affects health and weight. Discuss sleep hygiene strategies. |
| 8 | Taking Charge: Eating and Activity Cues | Discuss triggers/cues for eating and physical activity. Discuss method to modify problematic eating and activity cues. |
| 9 | An Introduction to Problem Solving | Discuss steps in Problem Solving model and how to apply them to current and future problems. |
| 10 | Talking Back to Negative Thoughts | Discuss common weight-related negative thoughts and patterns. Demonstrate how to “talk back” to negative thoughts. |
| 11 | Slipping but not Falling: Preventing Relapse | Identify risk factors for experiencing a slip. Create a plan to prevent slips from becoming a relapse. |
| 12 | Coping with High-Risk Situations and Eating Out the Healthy Way | Identify high-risk situations, challenges faced, and coping strategies. Demonstrate how to budget calories for eating out. |
| 13 | Social Support and Making Social Cues Work for You | Discuss strategies for building social support and ways to effectively manage social cues. |
| 14 | Managing Stress | Discuss causes and effects related to stress. Introduce healthy strategies for stress reduction. |
| 15 | Having a Positive Body Image and Dealing with Plateaus | Discuss facts about body image and plateaus. Review tools for overcoming plateaus. |
| 16 | Looking Forward: Planning Ahead | Review current progress and discuss future goals. |
| 17 | Phase II Program Goals: Food Tracking Review | Review positive changes made, goal setting, and importance of accurate food tracking. |

| | | |
|---|---|---|
| 18 | Tools for More Effective Problem Solving | Review problem solving model and apply to a current problem. |
| 19 | Meal Mastery: Build a Better Meal | Review benefits of planning ahead for meals. Identify small changes that can reduce calories in a meal. |
| 20 | Time for Health | Discuss importance of budgeting time for exercise. Set goals and problem solve how to make time for activity. |
| 21 | Plateau Busting | Review information about plateaus. Discuss ways to address a plateau. |
| 22 | Travel Triumphantly! | Identify barriers to healthy eating and activity while traveling. |
| 23 | Nutrition and Exercise During Illness | Identify strategies for overcoming illness by modifying physical activity and incorporating nutritious foods. |
| 24 | Super Sneakers, Stretching, and Safety | Identify appropriate footwear, clothing, and stretching techniques for physical activity. |
| 25 | Nutrition Myth Busters | Identify myths related to nutrition and healthy eating. |
| 26 | Planning Challenge: Perfectly Prepared | Review 7-day meal planning worksheet and strategies to prepare meals at home during busy times. |
| 27 | Planning Challenge: Food Swaps | Identify lower calorie substitutes for high calorie food, beverages, and ingredients. |
| 28 | Fitness Challenge: Hike the Himalayas | Identify barriers preventing planned exercise and problem solving strategies to overcome them. |
| 29 | Preventing Chronic Disease | Identify lifestyle changes that could prevent chronic diseases and problem solve strategies to implement them. |
| 30 | Mind Challenge: Empowering Emotions | Discuss how to positively manage emotions and review ways to relieve stress. |
| 31 | Life Lessons: Renewing Your Vows | Identify ways to overcome stress and maintain positive changes. Identify ways to modify goals and stick to them during stressful times. |
| 32 | Life Lessons: Reaching New Heights | Discuss strategies to keep healthy eating and physical activity exciting. |
| 33 | Mastering Maintenance and Continuing Goals | Review strategies from National Weight Control Registry. Discuss SMART goals. |
| 34 | Reflection and Planning for the Next Step | Reflect on healthy lifestyle changes made throughout the program. Review the importance of self-monitoring and problem solving. |
