# Supplementary material for: Effect of Telehealth Extended Care for Maintenance of Weight Loss in Rural US Communities: A Randomized Clinical Trial
Source: JAMA Netw Open. 2020 Jun 15;3(6):e206764. doi: 10.1001/jamanetworkopen.2020.6764 (PMC7296388; doi:10.1001/jamanetworkopen.2020.6764)
Supplement: Supplement 2. — Data Sharing Statement [file jamanetwopen-3-e206764-s002.pdf]

Data Sharing Statement

Perri. Effect of Telehealth Extended Care for Maintenance of Weight Loss in Rural US Communities. *JAMA Netw Open*. Published June 15, 2020. 10.1001/jamanetworkopen.2020.6764

Data
Data available: Yes
Data types: Deidentified participant data, Data dictionary
How to access data: Requests for data should be sent to Michael G. Perri; email address is mperri@ufl.edu
When available: With publication

Supporting Documents
Document types: None

Additional Information
Who can access the data: Data will be made available to researchers whose proposed use of the data has been approved.
Types of analyses: Systematic reviews and meta-analyses
Mechanisms of data availability: The data will be made available with investigator support after approval of a proposal and a signed data access agreement.
